# CD40-Mediated Activation of the NF-κB2 Pathway

**DOI:** 10.3389/fimmu.2013.00376

**Published:** 2013-11-15

**Authors:** Bruce S. Hostager, Gail A. Bishop

**Affiliations:** ^1^Department of Pediatrics, The University of Iowa, Iowa City, IA, USA; ^2^The Graduate Program in Immunology, The University of Iowa, Iowa City, IA, USA; ^3^Department of Microbiology, The University of Iowa, Iowa City, IA, USA; ^4^Department of Internal Medicine, The University of Iowa, Iowa City, IA, USA; ^5^Iowa City VA Medical Center, Iowa City, IA, USA

**Keywords:** CD40, NF-κB, signal transduction

## Abstract

CD40 is a critical stimulatory receptor on antigen-presenting cells of the immune system. CD40-mediated activation of B cells is particularly important for normal humoral immune function. Engagement of CD40 by its ligand, CD154, on the surface of activated T cells initiates a variety of signals in B cells including the activation of MAP kinases and NF-κB. The transcriptional regulator NF-κB is in reality a family of factors that can promote B cell activation, differentiation, and proliferation. Complex – and only partially understood – biochemical mechanisms allow CD40 to trigger two distinct NF-κB activation pathways resulting in the activation of canonical (NF-κB1) and non-canonical (NF-κB2) NF-κB. This brief review provides a summary of mechanisms responsible for activation of the latter, which appears to be particularly important for enhancing the viability of B cells at various stages in their life cycle and may also contribute to the development of B cell malignancies. CD40 is also expressed by various cell types in addition to B cells, including T cells, macrophages, dendritic cells, as well as certain non-hematopoietic cells. Here too, while perhaps less extensively studied than in B cells, the CD40-mediated activation of NF-κB2 also appears to have important roles in cellular physiology.

The transcriptional regulator NF-κB participates in many important activation events in B cells, including B cell proliferation and differentiation in response to signaling by tumor necrosis factor receptor (TNFR) family members or toll-like receptor (TLR) proteins (1). NF-κB is not a single transcription factor, but rather a family of factors composed of homo- and hetero-dimers of p50, p52, c-Rel, RelA (p65), and RelB. Activation of these dimer pairs occurs via two general mechanisms, sometimes referred to as the canonical and non-canonical pathways. Canonical (NF-κB1) activation is typically rapid as it does not usually require new protein synthesis. Activation of this pathway is mediated largely by degradation of inhibitors present in the cytoplasm, which allows transit of NF-κB dimers (typically p50/RelA or p50/c-Rel hetero-dimers) into the nucleus. In contrast, activation of non-canonical NF-κB (NF-κB2) often occurs after activation of the canonical pathway, may require new protein synthesis, and ultimately results in the nuclear localization of predominantly p52/RelB hetero-dimers, although p52/p65 and p52/c-Rel hetero-dimers have also been described (2). Excellent recent reviews discuss current general models of NF-κB2 activation (2–5). Briefly, the activation of the NF-κB2 pathway is largely regulated by the production and posttranslational processing of its precursor, p100. Its proteolytic processing is regulated by IκB kinase α (IKKα), which is in turn regulated by NF-κB-inducing kinase (NIK).

## CD40-Mediated NF-κB2 Activation in B Cells

CD40 signaling in B cells can modulate the activation of NF-κB2 at several points. First, CD40 signaling strongly activates NF-κB1 in B cells. This activation leads to the enhanced production of p100 (4). In addition, CD40 can regulate the posttranslational processing of p100 by regulating the activity of NIK (6). In resting cells, NIK activity is inhibited by a protein complex that includes TNFR-associated factor (TRAF)2, TRAF3, and cellular inhibitors of apoptosis (cIAP)1/2 (5). The major function of this complex appears to be in mediating the ubiquitination and degradation of NIK in resting B cells. Within the complex, TRAF3 appears to interact with NIK, while TRAF2 mediates interactions between TRAF3 and the cIAP molecules. Engagement of CD40 by its ligand leads to recruitment of TRAF proteins, including TRAFs 2 and 3, to the cytoplasmic domain of CD40. This event disrupts the NIK regulatory function of the TRAF2/TRAF3/cIAP complex, perhaps by promoting the ubiquitination, and degradation of TRAF3. With disruption of the NIK regulatory complex, NIK begins to accumulate (via new protein synthesis) in the cytoplasm to levels where it promotes the phosphorylation and activation of IKKα. In turn, IKKα activity mediates the phosphorylation of p100, which targets the protein for processing by the proteasome, resulting in p52 production (3–5).

A number of genetically modified mouse strains and cell lines have contributed to our understanding of NF-κB2 regulation by CD40 in B cells (7–11). TRAF2, TRAF3, and TRAF6 all significantly contribute to this CD40 signal transduction. Mouse B cell lines deficient in TRAF2 or TRAF6 exhibit little or no defect in CD40-mediated NF-κB2 activation (10). However, B cell lines doubly deficient in TRAF2 and TRAF6 appear defective in the activation of NF-κB2, suggesting that the two molecules have overlapping functions in the activation of this pathway. Potentially, these results are explained by the ability of CD40, through TRAF2 and TRAF6, to activate the NF-κB1 pathway, which in turn activates p100 transcription. Primary B cells from TRAF2-deficient mice exhibit elevated basal NF-κB2 activation (not observed in the cell line studies), which is only weakly augmented by CD40 signaling (9), consistent with a role for TRAF2 in the NIK regulatory complex. In the cell line studies, TRAF2 deficiency did not appear to augment the basal level of p52 production (10), although this may have been due to limited production of p100 in non-activated cells. B cells from mice conditionally deficient in TRAF3 specifically in B cells also exhibit strong constitutive activation of the NF-κB2 pathway (7, 11), which is again consistent with the model in which TRAF3 is a major component of the NIK regulatory complex. While the phenotypes of TRAF2-, cIAP-, and TRAF3-deficient primary B cells are somewhat similar with respect to the constitutive activation of NF-κB2, there are instructive differences between the strains [reviewed in (5)]. CD40-mediated differentiation of B cells into germinal center B cells is augmented in TRAF3-deficient mice (7, 11), but TRAF2- or cIAP-deficient mice have a significant defect in germinal center B cell development (8). This likely reflects the role of TRAF2/cIAP in activation of the NF-κB1 pathway, which TRAF3 does not share.

Interestingly, in B cell-specific TRAF3-deficient mice, the elevated constitutive activation of NF-κB2 can be enhanced somewhat by CD40 stimulation (11), indicating the existence of TRAF3-independent mechanisms of NIK/NF-κB2 regulation. This possibility is further supported by the observation that CD40 signaling can activate NF-κB2 in TRAF3-deficient B cell lines reconstituted with a mutant TRAF3 molecule that binds NIK robustly, but is not degraded following CD40 stimulation (Lin et al., this issue). These observations demonstrate that the regulation of NF-κB2 by CD40 in B cells is only partially understood. Recently, the protein kinase TANK-binding kinase 1 (TBK1) was shown to negatively regulate NF-κB2 activation and IgA Ig isotype switching in primary B cells, potentially through phosphorylation/degradation of NIK (12). The de-ubiquitinating enzyme OTUD7B was also shown recently to potentially negatively regulate NF-κB2 (and primary B cell activation) by inhibiting activation-induced degradation of TRAF3 (13). Additionally, zinc finger protein 91 has recently been shown to promote NIK stability in a CD40-stimulated epithelial tumor cell line, perhaps through K63-linked polyubiquitination of NIK (14). Whether this mechanism is relevant to the CD40-mediated activation of B cells remains to be demonstrated. It is likely that additional regulatory mechanisms will come to light in the next few years, and will contribute to our understanding of normal B cell biology as well as the physiology of B cell malignancies.

## CD40-Mediated NF-κB2 Activation in Non-B Cells

Compared to the extensive research performed in B cells, a cell type in which NF-κB activation is a major regulatory pathway, relatively little investigation has been performed on CD40-mediated NF-κB2 signaling in other cell types. Most studies of CD40 functions in non-B cells do not examine NF-kB activation. In those that do, canonical NF-κB1 activation is typically the focus, and/or methods used (reporter gene activation or electrophoretic mobility shift assay, EMSA) do not allow results to distinguish between NF-κB1 and NF-κB2 induction. We summarize below the information that is currently available on CD40-mediated induction of the NF-κB2 pathway in non-B cells.

### Other hematopoietic cells

#### T lymphocytes

The physiologic importance of direct signaling to T cells by CD40 has at times been a controversial topic (15). However, there is no doubt that normal activated CD4^+^ and CD8^+^ T cells can express CD40, and it appears to play significant biological roles in mouse models of T cell-dependent immune responses (16, 17), including autoimmune responses [reviewed in Ref. (15)]. However, the role of CD40-mediated NF-κB activation, and in particular NF-κB2 activation in these roles is unclear, and may be context-dependent. CD3^+^CD8^+^CD40^+^ T cells in Balb/c mice exhibit cytotoxic activity toward CD4^+^CD25^+^ T regulatory cells during *Leishmania* infection – this activity is CD40-dependent, but unaffected by inhibitors of NF-κB (18). A CD4^+^ mouse T cell line stably expressing transfected CD40 utilizes CD40 as an effective co-stimulatory signal with T cell receptor signals to activate T cell functions. In this model, both NF-κB1 and NF-κB2 pathways in T cells are activated by CD40 (19). The ultimate biological importance of NF-κB2 activation in T cell CD40 signaling remains to be explored.

#### Myeloid cells

Monocytes and macrophages, like B cells, constitutively express CD40, which activates mitogen-activated protein (MAP) kinases and NF-κB in these cells [reviewed in Ref. (20)]. However, very little is known about the potential involvement of the NF-κB2 pathway in macrophage CD40 signaling. Revy et al. performed a direct comparison between human monocytes and B cells activated through CD40. EMSA demonstrated that nuclear translocation of NF-κB binding activity is induced by CD40 in both cell types, and the p50 subunit involved in NF-κB1 activation is part of these nuclear complexes. Interestingly, the p65 subunit was only present in complexes from B cells (21). However, as the CD40 stimulation was only of short duration in these experiments (30 min); the activation of NF-κB2 was not assessed at later times.

CD40 also delivers potent and important signals to dendritic cells (DC) [reviewed in Ref. (22)]. Following signals from CD40 that stimulate the ability of DC to cross-present antigen to T cells, the nuclear translocation of p52 characteristic of NF-κB2 signaling is seen. Mice bearing a mutant NIK that disrupts NF-κB2 activation by various signals, the NIK^aly/aly^ mouse, show DC functional defects (23). CD40 fails to induce cross presentation of antigens to CD8 T cells in the NIK^aly/aly^ mouse, implicating NF-kB2 in this particular DC function. However, the evidence is indirect, as the NIK mutation is expressed in all cells of the mouse, and will impact multiple pathways of cell development and function. In another experimental model, human monocyte-derived DC treated with siRNA for NIK or IKKα are unable to produce the cytokines IDO and IL-6 following *in vitro* stimulation with the CD40 ligand, CD154. This also renders the treated DC unable to promote the induction of T regulatory cells (24). Thus, NF-κB2 signaling plays important roles in CD40 functions in DC.

### Non-hematopoietic cells

#### Epithelial cells

CD40 was first described as an antigen expressed on the surface of bladder carcinoma cells (25), and can be expressed on a variety of types of epithelial cells under particular circumstances [reviewed in (26, Lin et al., this issue)]. Unfortunately, by far the most commonly used cells of epithelial origin used to study CD40 signaling are the transformed cell lines HEK 293 and HeLa. In the majority of studies using these cell lines, CD40 and/or the signaling proteins studied are exogenously expressed at highly non-physiologic levels. Thus, because such models can provide misleading results [recently reviewed in (27)], we will not discuss this body of work in this article. Very little is known about the usage of the NF-κB2 pathway by endogenous CD40 expressed by normal epithelial cells. An interesting report using airway epithelial cells, which produce the cytokine RANTES upon CD40 stimulation, examined NF-κB activation by EMSA. Nuclear binding complexes for probes with sites characteristic of both NF-κB1 and NF-κB2 were identified, with the former peaking at 30 min of stimulation, and the latter at 2 h. The p65 subunit was present in both types of complexes (28). The functional importance of this NF-κB2 activation remains to be defined.

#### Fibroblasts

Although CD40 can be expressed by fibroblasts, no information is currently available on the extent or importance of NF-κB2 activation by CD40 in this cell type. A recent report made the interesting observation that TWEAK, a molecule implicated in various fibrotic processes [reviewed in (29)] inhibits CD40 signaling in the HeLa cell line (30). However, neither fibroblasts nor NF-κB2 pathways were examined in this study.

#### Vascular endothelium

It is well-known that CD40 can be expressed on activated endothelium, and its interaction with CD154 has been implicated in the pathogenesis of atherosclerosis [reviewed in Ref. (31)]. A specific role for NF-κB2 activation has yet to be explored in this CD40 function. In human vascular endothelial cells, stimulation of CD40 with soluble CD154 stimulates the phosphorylation and nuclear translocation of the p65 subunit of NF-κB at 6 h (32). However, it is unclear whether this represents activation of the NF-κB2 pathway.

While it is clear that the mechanisms of CD40-mediated NF-κB activation are complicated (and still incompletely characterized), the consequences of NF-κB activation in B cells and other cells are orders of magnitude more complex. Many of the genes regulated by NF-κB have been identified by experiment and by computer analysis, but the work of understanding the subsequent roles of these genes and their interactions in cellular physiology is only in its infancy. Nevertheless, continued characterization of NF-κB activation mechanisms and consequences of NF-κB-regulated transcription will lead to a more complete understanding of basic immune system biology as well as the identification of molecular events and interactions that can be exploited in the treatment of immune disorders and cancer.

## Conflict of Interest Statement

The authors declare that the research was conducted in the absence of any commercial or financial relationships that could be construed as a potential conflict of interest.
